# Pregnancy Termination and Postnatal Major Congenital Heart Defect Prevalence After Introduction of Prenatal Cardiac Screening

**DOI:** 10.1001/jamanetworkopen.2023.34069

**Published:** 2023-09-15

**Authors:** Viktor Tomek, Hana Jičínská, Jan Pavlíček, Jan Kovanda, Petr Jehlička, Eva Klásková, Jiří Mrázek, David Čutka, Dagmar Smetanová, Miroslav Břešťák, Pavel Vlašín, Markéta Pavlíková, Václav Chaloupecký, Jan Janoušek, Jan Marek

**Affiliations:** 1 Children’s Heart Centre, Second Faculty of Medicine, Motol University Hospital, Charles University, Prague, the Czech Republic; 2 Department of Pediatric Cardiology, The University Hospital Brno, Faculty of Medicine of Masaryk University, Brno, the Czech Republic; 3 Department of Pediatrics and Prenatal Cardiology, University Hospital Ostrava, Ostrava, the Czech Republic; 4 Department of Pediatrics, University Hospital in Pilsen, Charles University, Pilsen, the Czech Republic; 5 Department of Pediatrics, Olomouc University Hospital and Faculty of Medicine and Dentistry, Palacký University Olomouc, Olomouc, the Czech Republic; 6 Department of Pediatrics, Masaryk Hospital, Ústí nad Labem, the Czech Republic; 7 Centre for Medical Genetics, České Budějovice, the Czech Republic; 8 Gennet, Centre for Fetal Medicine and Reproductive Genetics, Prague, the Czech Republic; 9 Department of Obstetrics and Gynecology of the First Faculty of Medicine, Charles University and General University Hospital, Prague, the Czech Republic; 10 Fetal Medicine Center, Brno, the Czech Republic; 11 Department of Probability and Mathematical Statistics, Faculty of Mathematics and Physics, Charles University, Prague, the Czech Republic; 12 Great Ormond Street Hospital for Children and Institute of Cardiovascular Sciences UCL, London, United Kingdom

## Abstract

**Importance:**

Prenatal cardiac screening of the first and second trimesters has had a major impact on postnatal prevalence of congenital heart defects (CHDs), rates of termination of pregnancy (TOP), and outcomes among children born alive with CHDs.

**Objective:**

To examine the prenatal and postnatal incidence of major CHDs (ie, necessitating intervention within the first year of life), detection rate trends, rates of TOP, and the association of cardiac screening with postnatal outcomes.

**Design, Settings, and Participants:**

In this cross-sectional study, 3827 fetuses with antenatally diagnosed major CHDs in the Czech Republic (population 10.7 million) between 1991 and 2021 were prospectively evaluated with known outcomes and associated comorbidities. Prenatal and postnatal prevalence of CHD in an unselected population was assessed by comparison with a retrospective analysis of all children born alive with major CHDs in the same period (5454 children), using national data registry. Data analysis was conducted from January 1991 to December 2021.

**Main Outcomes and Measures:**

Prenatal detection and postnatal prevalence of major CHDs and rate of TOPs in a setting with a centralized health care system over 31 years.

**Results:**

A total of 3 300 068 children were born alive during the study period. Major CHD was diagnosed in 3827 fetuses, of whom 1646 (43.0%) were born, 2069 (54.1%) resulted in TOP, and 112 (2.9%) died prenatally. The prenatal detection rate increased from 6.2% in 1991 to 82.8% in 2021 (*P* < .001). Termination of pregnancy decreased from 70% in 1991 to 43% (*P* < .001) in 2021. Of 627 fetuses diagnosed in the first trimester (introduced in 2007), 460 were terminated (73.3%). Since 2007, of 2066 fetuses diagnosed in the second trimester, 880 (42.6%) were terminated, resulting in an odds ratio of 3.6 (95% CI, 2.8-4.6; *P* < .001) for TOP in the first trimester compared with the second trimester. Postnatal prevalence of major CHDs declined from 0.21% to 0.14% (*P* < .001). The total incidence (combining prenatal detection of terminated fetuses with postnatal prevalence) of major CHD remained at 0.23% during the study period.

**Conclusions and Relevance:**

In this cross-sectional study, the total incidence of major CHD did not change significantly during the 31-year study period. The prenatal detection of major CHD approached 83% in the current era. Postnatal prevalence of major CHD decreased significantly due to early TOPs and intrauterine deaths. The introduction of first trimester screening resulted in a higher termination rate in the first trimester but did not revert the overall decreasing trend of termination for CHDs in general.

## Key Points

**Question:**

Is centralized prenatal cardiac screening associated with the postnatal prevalence of congenital heart defects (CHD), and was postnatal prevalence affected by the introduction of the first trimester screening?

**Findings:**

In this cross-sectional study of more than 3.3 million children, the combined prenatal and postnatal incidence of major CHDs remained unchanged over the 3 decades studied. The introduction of first trimester screening resulted in a higher termination of pregnancy (TOP) rate of fetuses with univentricular heart and those with associated comorbidities at an early stage but did not revert the overall decreasing trend in TOP.

**Meaning:**

In this study, the TOP rate decreased significantly for cardiac anomalies with favorable outcome and has become uncommon in recent years.

## Teaser

This cross-sectional study examines prenatal and postnatal incidence of major congenital heart defects (CHDs), detection rate trends, and the association of cardiac screening with postnatal outcomes.

## Introduction

Congenital heart defects (CHDs) are the most frequent congenital developmental anomalies, affecting from 0.6% to 2.2% of live-born children depending on the demographics and the study period.^1,2,3^ The birth prevalence of CHD shows variation, raising questions about time trends in CHD incidence.^4,5^ The occurrence of major CHD in children is affected by several variables. Since the introduction of fetal ultrasound scans and biochemical marker assessment, postnatal incidence has been informed by various approaches to prenatal screening worldwide, ranging from limited to well-organized fetal screening programs, leading to different detection rates of CHDs.^6,7^ Some studies have demonstrated that ethnicity, lower maternal education, worse access to health insurance, and lower socioeconomic status are associated with lower prenatal detection rates within the same delimited area.^8,9^ Several studies have confirmed gradual increases in the fetal detection rate, but only a few were designed as nationwide studies.^10,11,12^ Prenatal diagnosis may provide parents with the choice of termination of pregnancy (TOP) based on the expected outcome of severe CHD learned of during fetal counselling.^13,14^ High rates of TOP combined with fetal demise reduce the number of babies born alive with CHDs and make it difficult to determine the true CHD incidence.^15^ In our previous study following the implementation of regional first trimester screening in the Czech Republic, we found a significant association between a fetal cardiac screening program and postnatal outcomes due to a high detection rate of severe forms of CHD and significant comorbidities, resulting in an increased TOP rate in the first trimester.^16^ In this study, we aim to evaluate whether the increased TOP rate in the first trimester is associated with the rate of TOPs with major CHDs in nationwide prenatal screening.

## Methods

### Study Design and Participants

A total of 3 300 068 children were born alive in the Czech Republic (population 10.7 million) between 1991 and 2021. During the 31-year study period, a total of 3827 fetuses with antenatally diagnosed major CHD were prospectively evaluated with known outcomes and associated comorbidities. Prenatal and postnatal prevalence of CHD in an unselected population was assessed by comparison with a retrospective analysis of all children born alive with major CHDs in the same period (5454 children), using national data registry. This study was a registry research study and was approved by the institutional review board of the University Hospital Motol. Informed consent was waived, as the data analysis was performed retrospectively and anonymously from compulsory enrollment to the National Fetal Database of CHD. This study followed the Strengthening the Reporting of Observational Studies in Epidemiology (STROBE) reporting guideline.

### Prenatal Study Group

In the Czech Republic, the prenatal screening program includes 2 ultrasound scans covered by the basic national health insurance, during the 1st trimester (11th-14th week of gestation) and the 2nd trimester (18th-22nd week), and biochemical screening (PAPP-A and free ß-HCG in the first trimester; HCG, AFP, and uE3 in the second trimester). The first trimester screening with basic heart anatomy assessment was introduced in 2007.^17^ Both screenings include examination of 4-chamber view and outflow tracts; identification of visceroatrial situs, systemic, and pulmonary venous connections; 3-vessel and trachea view; and sagittal views of the aortic and ductal arches. Use of color flow Doppler in routine screening was encouraged. Pregnancies referred by fetal medicine specialists and those classified as high risk for CHD according to international guidelines^18^ were referred to local prenatal cardiologist for further review to eliminate false positives.^19^

Pregnancies suspected of CHDs were referred to 2 specialized centers for completion of final diagnosis and family counseling. All fetal cardiac diagnoses were confirmed in newborns or postmortem in deceased children or fetuses (compulsory by law). All patient data were stored in the National Fetal Database of CHD administered by the National Health Information System since its foundation in 1981. It is compulsory for all parties involved in prenatal screening or specialized fetal assessment to contribute to the National Fetal Database of CHD, thus limiting any loss of data.

### Postnatal Study Group

Every child born with suspected heart disease is examined by local pediatric cardiologists or neonatologists trained in cardiology. If major CHD is suspected, the child is referred to the single heart center for further evaluation and treatment. Numbers of CHDs were derived from the heart center database and verified by the National Health Information System registry on screening in the Czech Republic.^20^ A comparison of prenatal and postnatal incidence of CHD was considered reliable due to the national organization of health care, minimal migration, and the low neonatal mortality rate.

### Definition of Diagnosis

Cardiac and extracardiac malformations were classified using the World Heath Organization *International Statistical Classification of Diseases and Related Health Problems, Tenth Revision (ICD-10)*. Major CHDs were defined as malformations of the heart and great arteries that usually necessitate intervention within the first year of life.^19^ All fetal and postnatal CHD diagnoses were classified and listed in groups according to the most important lesions, as follows^21^: (1) tricuspid atresia, (2) mitral atresia, (3) double inlet ventricle, (4) congenitally corrected transposition of the great arteries, (5) hypoplastic left heart syndrome, (6) persistent truncus arteriosus, (7) transposition of the great arteries, (8) interrupted aortic arch, (9) atrioventricular septal defect, (10) double outlet right ventricle, (11) coarctation of the aorta, (12) absent pulmonary valve syndrome, (13) Ebstein anomaly, (14) pulmonary atresia, (15) tetralogy of Fallot, and (16) total anomalous pulmonary venous connection.

### Statistical Analysis

Total incidence was calculated as the number of major CHDs detected after birth plus the number of terminated pregnancies with major CHDs divided by the total number of live-born children plus pregnancies terminated in utero after 12 completed weeks of gestation. Detection rates were calculated as the number of prenatally detected fetuses with a major CHD divided by the number of live-born children with a major CHD plus fetuses with major CHD dying in utero and terminated pregnancies with major CHD, presented as percentages. We calculated 95% CIs for overall incidence rate estimates using an exact binomial test procedure.

As the annual data for some rare CHDs can be sparse and detection rates based on annual data can vary significantly, we used 5-year moving rates. Data in both the numerator and the denominator were summed over a 5-year period. In the figures, such results were plotted at the upper end point of the 5-year interval.

Gradual changes in total incidence, the prenatal detection rate, and postnatal prevalence as well as incidences and detection rates of individual CHDs were assessed using linear regression models. In the text, *P* values correspond to the *t* test for the regression coefficient.

The association of noncardiac anomalies with the decision to terminate the pregnancy after prenatal CHD diagnosis was explored using Fisher exact test and odds ratio (OR) calculated as odds of TOP in cases with a noncardiac anomaly divided by those without anomalies. All differences in frequencies between groups of patients were tested by the Fisher exact test. *P* < .05 was considered significant. All analyses were performed using statistical language and environment R version 4.1.0 (R Foundation for Statistical Computing).

## Results

### CHD Prevalence

**Figure 1.**
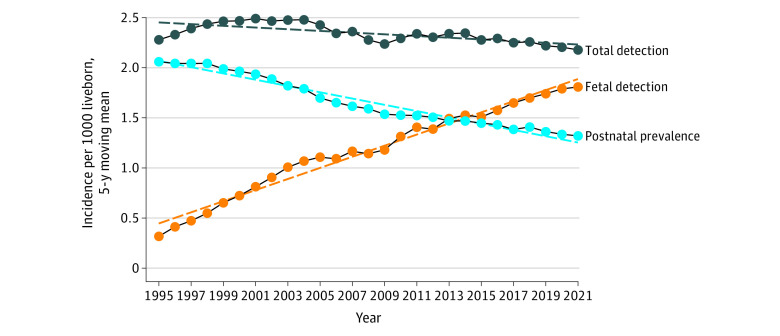
Total Incidence, Fetal Detection, and Postnatal Prevalence of Major Congenital Heart Defects by Year Overall trends estimated by linear regression models. Five-year moving rates are displayed, and results are plotted at the upper end point of the 5-year interval.

From 1991 to 2021, a total of 3 300 068 children were born alive, and 51 812 patients were examined in a single heart center with the diagnosis of a heart anomaly. Major CHDs were diagnosed in 5454 children. Postnatal prevalence of major CHD declined from a maximum of 2.02 per 1000 live-born children in 1995 to 1.35 per 1000 live-born children in 2021, a mean decline of 0.03 cases per 1000 live-born children per year (*P* < .001) (Figure 1).

CHD was observed in 5625 fetuses, of whom 3827 were diagnosed with a major heart defect and 1798 with less severe heart anomalies. Less important CHDs, such as aortic and pulmonary stenosis, atrial and ventricular septal defects and vascular rings, although some required surgery at an early age, were not included in the study due to variable prenatal and postnatal natural history. Of the 3827 fetuses with major CHDs, 1646 (43.0%) were born, 2069 (54.1%) were terminated, and 112 (2.9%) died in utero. The average detected prenatal incidence of CHD was 1.11 per 1000 live-born children, increasing from 1.30 per 1000 live-born children in 1991 to 1.78 per 1000 live-born children in 2021, representing a growth of 0.058 cases per 1000 live-born children per year (*P* < .001) (Figure 1). The gestational age declined from 23 weeks in 1991 to 16 weeks in 2021 (*P* < .001), with median (range) detection at 18 (11-41) weeks.

**Figure 2.**
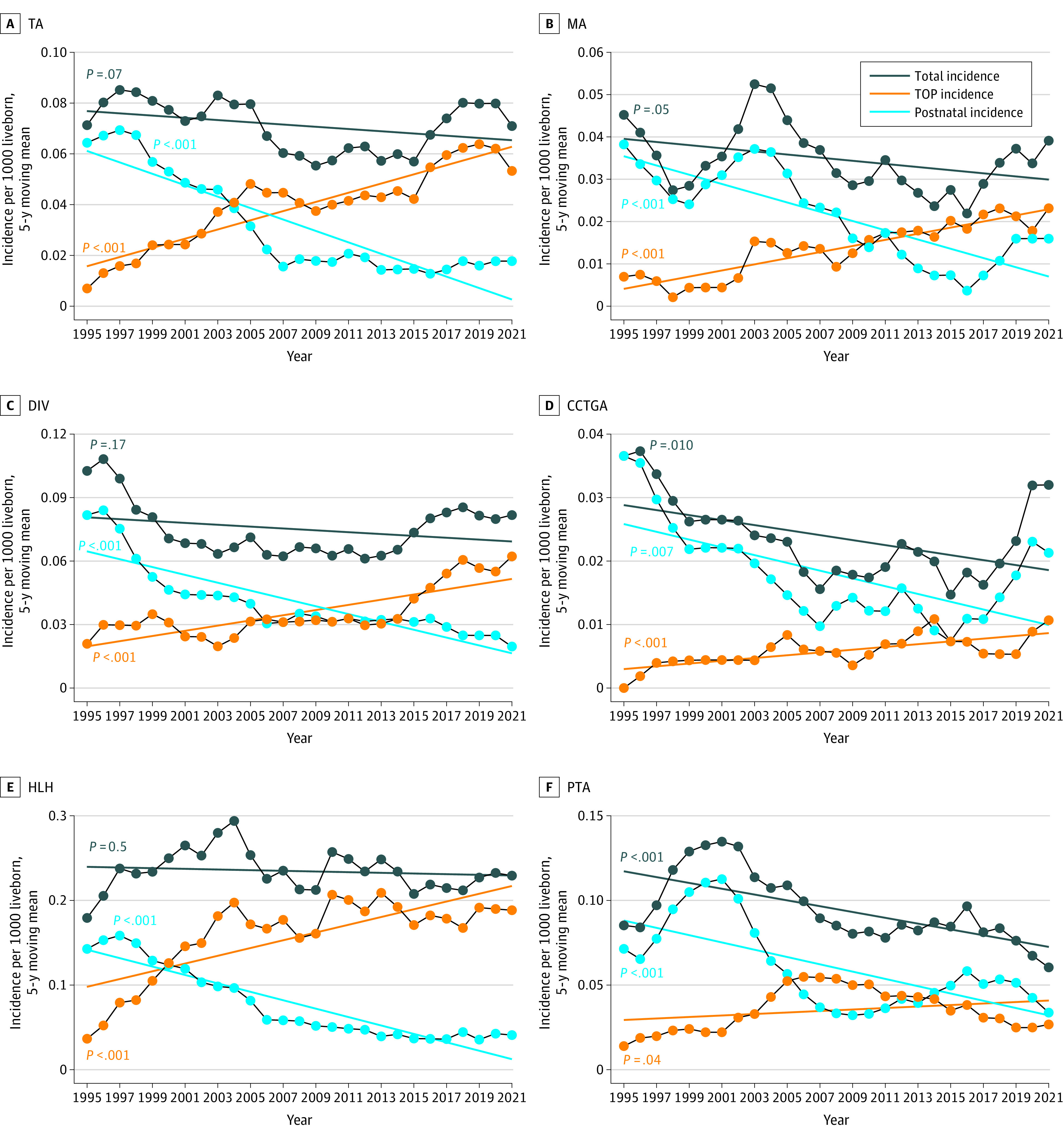
Total Incidence, Termination Rate, and Postnatal Incidence of Individual Congenital Heart Defect by Year Five-year moving rates are displayed. Results are plotted at the upper end point of the 5-year interval. Overall trends estimated by linear regression models. CCTGA indicates congenitally corrected transposition of the great arteries; DIV, double inlet ventricle; HLH, hypoplastic left heart; MA, mitral atresia; PTA, persistent truncus arteriosus; TA, tricuspid atresia; and TOP, termination of pregnancy.

Total incidence of CHD was 2.3 (95% CI, 2.28-2.39) per 1000 live-born children, with a very slight decline of −0.0034 cases per 1000 live-born children per year (*P* = .32) (Figure 1). The incidence of individual heart lesions is summarized in Figure 2 and in eFigure 1 and eFigure 2 in Supplement 1. There was a significant decrease in postnatal prevalence of heart lesions with the most complex CHDs such as tricuspid atresia, mitral atresia, double inlet ventricle, hypoplastic left heart syndrome, persistent truncus arteriosus, atrioventricular septal defect, pulmonary atresia, tetralogy of Fallot, congenitally corrected transposition of the great arteries, and double outlet right ventricle.

### Detection Rates

**Figure 3.**
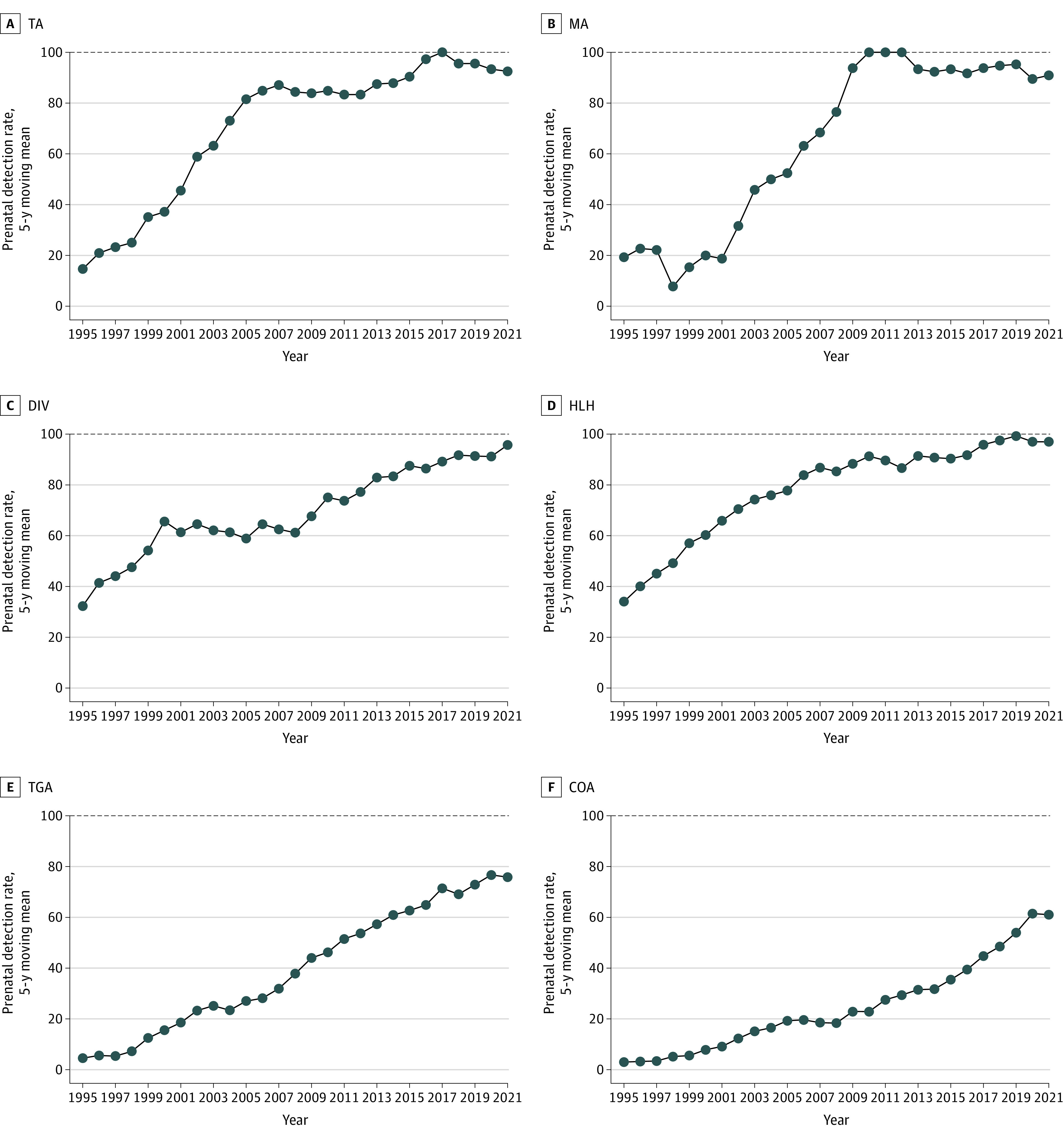
Prenatal Detection Rates of Individual Heart Defects Rates are based on a total number of prenatal detections (the numerator) and prenatal plus postnatal detections (the denominator) of the individual congenital heart defects. Five-year moving rates are displayed. Results are plotted at the upper end point of the 5-year interval to level individual variations in small numbers. CCTGA indicates congenitally corrected transposition of the great arteries; DIV, double inlet ventricle; HLH, hypoplastic left heart; MA, mitral atresia; PTA, persistent truncus arteriosus; and TA, tricuspid atresia.

During the 31-year interval, 50.1% of major CHDs were detected prenatally, increasing from 6.2% in 1991 to 82.9% in 2021 (*P* < .001) (eFigure 3 in Supplement 1). Detection rates of specific major CHDs appeared to progress during the study period. Detection rates of univentricular heart lesions (mitral atresia, tricuspid atresia, double inlet ventricle, hypoplastic left heart syndrome) varied between 71% and 100%. Initially low detection rates of coarctation of the aorta and transposition of the great arteries increased to 50% and 70%, respectively, in the last years (Figure 3). Detection rates of other specific major CHDs appear in eFigure 4 and eFigure 5 in Supplement 1.

An associated anomaly (chromosomal and structural) was found in 1113 of 3827 fetuses (29.1%) with major CHD. Chromosomal anomalies were detected in 811 (21.2%) and structural in 562 (14.7%). The most common chromosomal anomalies were trisomy 21 (269 of 811 [33.2%]), trisomy 18 (220 [27.1%]), deletions (182 [19.5%]), and Turner syndrome (98 [12.1%]). Most frequent noncardiac anomalies were detected among fetuses with atrioventricular septal defect (439 of 781 [56.2%), interrupted aortic arch (22 of 42[52.4%]), double outlet right ventricle (158 of 394 [40.1%]), persistent truncus arteriosus (56 of 146 [38.4%]), and tetralogy of Fallot (82 of 299 [27.4%]).

### Pregnancy Outcomes

**Figure 4.**
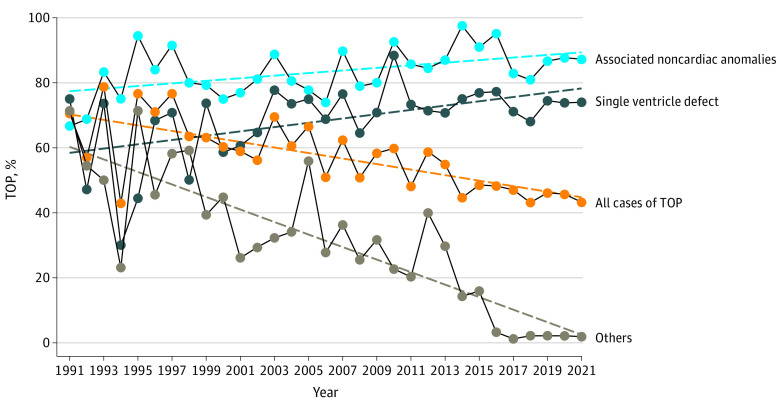
Rate of Termination of Pregnancy (TOP)

During the study period, 54.1% (95% CI, 52.2%-59.6%) of fetuses major CHDs resulted in TOPs based on the parent’s informed decision. The proportion of TOP decreased from 70% in 1992 to 43% in 2021 (*P* < .001). High termination rates were documented in fetuses with univentricular heart morphology (hypoplastic left heart syndrome, 500 of 583 [85.7%]; tricuspid atresia, 125 of 153 [80.1%]; double inlet ventricle, 124 of 179 [69.3%]; mitral atresia, 46 of 73 [63.0%]) and in CHD associated with a large proportion of noncardiac anomalies (persistent truncus arteriosus, 109 of 146 [74.6%]; atrioventricular septal defect, 529 of 781 [67.7%]; interrupted aortic arch, 28 of 42 [66.6%]; double outlet right ventricle, 239 of 394 [60.1%]). Additional noncardiac anomalies were associated with TOP in all cases with CHDs; the strongest of which was observed in heart lesions with otherwise usually favorable prognoses (ie, tetralogy of Fallot, coarctation of the aorta, atrioventricular septal defect, and transposition of the great arteries) (Table). The total number of TOPs for fetuses with univentricular heart morphology remained unchanged (0.10 cases per 1000 live-born children per year; *P* = .63) and the number of TOPs associated with noncardiac anomalies increased during the study period (0.51 cases per 1000 live-born children per year; *P* = .006). On the contrary, the rate of TOP for fetuses with isolated heart defects allowing for biventricular repair dropped from 61% in 1992 to an absolute minimum of 1% to 3% (*P* < .001) in the present time (Figure 4).

**Table.** Association of Extracardiac Malformations With TOP in Individual CHDs^a^

CHD	Associated noncardiac anomalies present		Associated noncardiac anomalies absent		OR (95% CI)^b^
	TOP, No.	Pregnancy continued, No.	TOP, No.	Pregnancy continued, No.	
TA	24	2	97	35	4.30 (0.98-39.45)
MA	13	1	31	28	11.45 (1.53-515.14)
DIV	26	4	98	53	3.50 (1.13-14.51)
CCTGA	3	0	17	25	NA (0.54-∞)
HLH	108	9	368	99	2.99 (1.44-6.96)
PTA	48	8	52	38	4.34 (1.77-11.88)
TGA	23	1	38	337	199.11 (30.66-7954.03)
IAA	20	2	11	14	12.01 (2.16-128.15)
AVSD	424	18	61	267	101.81 (58.36-186.81)
DORV	145	11	84	156	24.26 (12.29-52.55)
COA	51	11	7	254	159.58 (57.21-515.79)
PVLVABS	2	0	14	14	NA (0.17-∞)
EBST	11	1	18	66	38.53 (5.03-1743.28)
PA	54	4	127	81	8.56 (2.99-33.78)
TOF	75	7	11	210	193.77 (70.58-621.92)
TAPVC	1	0	2	8	NA (0.07-∞)

Abbreviations: AVSD, atrioventricular septal defect; CCTGA, congenitally corrected transposition of the great arteries; COA, coarctation of the aorta; DIV, double inlet ventricle; DORV, double outlet right ventricle; EBST, Ebstein anomaly; HLH, hypoplastic left heart; IAA, interrupted aortic arch; MA, mitral atresia; NA, not applicable; OR, odds ratio; PA, pulmonary atresia; PTA, persistent truncus arteriosus; PVLVABS, absent pulmonary valve syndrome; TA, tricuspid atresia; TAPVC, total anomalous pulmonary venous connection; TGA, transposition of the great arteries; TOF, tetralogy of Fallot; TOP, termination of pregnancy.

^a^ Summary data for years 1991 to 2018. Odds and respective ORs could not be established for rare events with 0 in the denominator.

^b^ OR and 95% CIs were rounded to 2 valid digits and represent the odds of termination for fetuses with vs without noncardiac anomalies.

### First Trimester Screening Outcomes 

Since the introduction of the first trimester cardiac screening in 2007, the proportion of diagnosed CHDs in the first trimester increased from 7.1% to 26.7% in 2013 (*P* < .001) and remained constant in subsequent years. Associated anomalies were identified 9.5 (95% CI, 7.5-11.9) times more often in first trimester diagnosis (401 of 627 [64.0%]) vs the second trimester (383 of 2066 [18.5%]) (*P* < .001). A single ventricle morphology was diagnosed more frequently in the first trimester (288 of 627 [46.0%]) than in the second trimester (386 of 2066 [18.7%]; OR, 3.6; 95% CI, 2.9-4.5; *P* < .001). Of 627 fetuses diagnosed in the first trimester, 30 (4.8%) died prenatally and 455 (72.6%) were terminated. Of 2066 fetuses diagnosed in the second trimester, 37 (1.8%) died and 833 (40.3%) were terminated, resulting in an odds ratio of 3.6 (95% CI, 2.8-4.6; *P* < .001) for TOP when comparing the first and the second trimester. An increasing number of associated noncardiac anomalies was identified during the first trimester screening (eFigure 6 in Supplement 1).

## Discussion

### Total Incidence and Prenatal and Postnatal CHD Prevalence

To our knowledge, this is the largest nationwide study of the association of the prenatal cardiac screening with postnatal outcomes in children born with CHDs. These prenatal prospective and postnatal retrospective analyses in the setting of centralized health care, national data registry, and compulsory postmortem assessment demonstrated an increasing prenatal detection rate of major CHDs and those associated with additional comorbidities. We found that the overall incidence of major CHDs did not change significantly during the study period and that the estimated total incidence of major CHDs of 2.3 per 1000 live-born children per year corresponded to the data from the previous study conducted by Šamánek et al^1^ in part of the Czech Republic during 1980 to 1990. Similar data were published in studies from Denmark and Norway.^10,15^ Other studies, particularly from Asia, have suggested that increased CHD incidence could be caused by increased environmental pollution and/or genetically due to higher consanguinity.^14,15^ Continuing improvements in postnatal detection may also be a factor,^4^ alongside higher proportion of genetic disorders associated with CHDs.^11^

An increase in total incidence was observed in several CHDs. The increasing incidence of atrioventricular septal defect and coarctation of the aorta could be explained by higher prenatal detection of fetuses that would not naturally survive childbirth, mainly due to associated extracardiac abnormalities rather than due to isolated heart failure.^22^

CHD prenatal detection rates and specifically those of major CHDs have increased dramatically over time, and high detection rates are consistent with similar recent reports.^10,21,23^ The change in expertise, ultrasonography equipment, and increased training of fetal specialists have contributed to improved fetal detection. However, the main improvement of prenatal detection rates was achieved by involving pediatric cardiologists specialized in fetal cardiac scanning in the screening program.^12^ Some studies differ in the method of detection rate determination. Quatermain et al^24^ used the Society of Thoracic Surgeons Congenital Heart Surgery database of children from 91 participating centers across the United States undergoing operations at younger than 6 months of life and analyzed the overall rate and spectrum of prenatally diagnosed heart lesions. The study showed an increasing detection rate, namely from 26% in 2006 to 42% in 2012.^24^ Similar methods were used in other studies comparing the number of prenatally detected heart defects with those known postnatally from a national patient registry.^10,15,19^

### First Trimester Cardiac Screening Program Implementation and Postnatal Outcomes

In our previous study following the implementation of regional first trimester screening in the Czech Republic, we found a significant association of fetal cardiac screening program with postnatal outcomes due to high detection rate of severe forms of CHDs and significant comorbidities, resulting in an increased TOP rate in the first trimester.^16^ The number of early terminations with single-ventricle morphology or those with noncardiac anomalies remained high; however, the rate of TOP for all other lesions significantly declined to 43% in 2021. Similar termination rates were documented in other studies (Lytzen et al,^10^ 39.1%; Khoshnood et al,^25^ 41%), with examples of even higher rates (Galindo et al,^26^ 61%).

However, the assumption that reduced numbers of children are born with CHDs due to early terminations remains speculative and ethically controversial. Our study suggests that it may rather be the result of increased detection of serious, particularly genetic, disorders identified in the first trimester followed by early termination without diagnosed CHDs. It could also be explained by increasing age of pregnant women (≥35 years); difficulties conceiving naturally, resulting in more in vitro pregnancies; and greater confidence in improving outcomes among children with CHDs.^6,27^

It has been well recognized that the primary diagnostic information about CHD given to parents plays an important role in their decision-making.^28^ That is why we have repeatedly urged gynecologists and fetal medicine specialists to closely collaborate with fetal cardiologists who are responsible for providing a definitive description of the cardiac anatomy and function and offering the family expert opinions on natural history, treatment options, and long-term postnatal outcomes. This course of action, along with ensuing optimism of improved outcomes, may also have influenced the parents’ decision.

Terminations due to fetal genetic conditions may occur even in pregnancies with negative combined screening and thus possibly without detection of CHDs.^29^ Prenatal detection rates still vary among major cardiac anomalies, ranging from 25% in total anomalous pulmonary venous connection to 97% in hypoplastic left heart syndrome in this study. Similar data were reported in the literature with detection rates up to 100% in cases with univentricular heart.^10^ We have observed a gradual increase in the detection of heart lesions with outflow tract anomalies, most likely due to improvement of screening by implementing the 3-vessel and trachea view.^30,31^ Fetal diagnoses of coarctation of the aorta and transposition of the great arteries have a significant impact on postnatal outcomes, but the diagnosis remains challenging.^24,32,33^ However, in our study, the detection rates of these defects increased in recent years following the implementation of the 3-vessel and trachea view according to the International Society of Ultrasound in Obstetrics and Gynecology guidelines.^34^

We recorded an overall decrease in the postnatal incidence of CHDs due to a high proportion of early terminations. Analogous changes in the proportion of CHDs have been described in other studies.^6,10,13,15^

### First Trimester Cardiac Screening Program Risks and Benefits

It was suggested by Persico et al^35^ that the vast majority of major CHDs can be detected during the first trimester fetal ultrasound scan if the scan is performed by experienced fetal sonographers. However, in our nationwide study, the first trimester screening, performed by likely less trained gynecologists, could identify only 27% of all prenatally detected major CHDs. The first trimester diagnosis of CHD showed high sensitivity and specificity for the detection of any cardiac anomaly but poor predictive value of lesion’s severity due to the natural progression of some types of defects over time.^36^ This emphasizes the role of experienced fetal specialists in avoiding incorrect conclusions based on inappropriate diagnosis or its severity, such as the problematic differential diagnosis of small left ventricle in isolated coarctation of the aorta vs hypoplastic left heart syndrome.

### Limitations

Despite the low neonatal mortality rate (in the last years of the study, <0.15% as documented by The National Health Information System^20^), it is not possible to rule out all postnatally deceased children with unrecognized CHDs. According to the Czech National Statistical Information System,^37^ migration is minimal, but it can still affect the accuracy of the data.

## Conclusions

In this cross-sectional study, the total incidence of major CHD did not change significantly during the 31-year study period. Nationwide prenatal detection of major CHD exceeded 80% in the current era. TOP in the case of major CHDs has decreased from 70% in 1991 to 43% in 2021 but remains high in fetuses with univentricular heart and those with major CHD–associated comorbidities. The introduction of first trimester screening resulted in a higher TOP rate at early stages but did not revert the overall decreasing trend in TOP. In cardiac anomalies with favorable outcomes, the TOP rate decreased significantly, becoming rather exceptional in recent years.

## Notes

Supplement 1. **eFigure 1.** Total Incidence, Termination Rate, and Postnatal Incidence of Ebstein Anomaly, Pulmonary Atresia, Tetralogy of Fallot, and Total Anomalous Pulmonary Venous Connection per Year **eFigure 2.** Total Incidence, Termination Rate, and Postnatal Incidence of Transposition of the Great Arteries, Interrupted Aortic Arch, Atrioventricular Septal Defect, Double Outlet Right Ventricle, Coarctation of the Aorta, Absent Pulmonary Valve Syndrome per Year **eFigure 3.** Total Prenatal Detection Rates of Major Congenital Heart Defects **eFigure 4.** Prenatal Detection Rates of Congenitally Corrected Transposition of the Great Arteries, Interrupted Aortic Arch, Atrioventricular Septal Defect, Double Outlet Right Ventricle, Ebstein Anomaly, and Pulmonary Atresia by Year **eFigure 5.** Prenatal Detection Rates of Persistent Truncus Arteriosus, Absent Pulmonary Valve Syndrome, Tetralogy of Fallot, and Total Anomalous Pulmonary Venous Connection **eFigure 6.** First Trimester Screening Period

Supplement 2.
Data Sharing Statement
