# Supplementary material for: Pregnancy Termination and Postnatal Major Congenital Heart Defect Prevalence After Introduction of Prenatal Cardiac Screening
Source: JAMA Netw Open. 2023 Sep 15;6(9):e2334069. doi: 10.1001/jamanetworkopen.2023.34069 (PMC10504618; doi:10.1001/jamanetworkopen.2023.34069)
Supplement: Supplement 1. — eFigure 1. Total Incidence, Termination Rate, and Postnatal Incidence of Ebstein Anomaly, Pulmonary Atresia, Tetralogy of Fallot, and Total Anomalous Pulmonary Venous Connection per Year eFigure 2. Total Incidence, Termination Rate, and Postnatal Incidence of Transposition of the Great Arteries, Interrupted Aortic Arch, Atrioventricular Septal Defect, Double Outlet Right Ventricle, Coarctation of the Aorta, Absent Pulmonary Valve Syndrome per Year eFigure 3. Total Prenatal Detection Rates of Major Congenital Heart Defects eFigure 4. Prenatal Detection Rates of Congenitally Corrected Transposition of the Great Arteries, Interrupted Aortic Arch, Atrioventricular Septal Defect, Double Outlet Right Ventricle, Ebstein Anomaly, and Pulmonary Atresia by Year eFigure 5. Prenatal Detection Rates of Persistent Truncus Arteriosus, Absent Pulmonary Valve Syndrome, Tetralogy of Fallot, and Total Anomalous Pulmonary Venous Connection eFigure 6. First Trimester Screening Period [file jamanetwopen-e2334069-s001.pdf]

Supplemental Online Content

Tomek V, Jičínská H, Pavlíček J. J, et al. Pregnancy termination and postnatal major congenital heart defect prevalence. *JAMA Netw Open.* 2023;6(9):e2334069.
doi:10.1001/jamanetworkopen.2023.34069

eFigure 1. Total Incidence, Termination Rate, and Postnatal Incidence of Ebstein Anomaly, Pulmonary Atresia, Tetralogy of Fallot, and Total Anomalous Pulmonary Venous Connection per Year

eFigure 2. Total Incidence, Termination Rate, and Postnatal Incidence of Transposition of the Great Arteries, Interrupted Aortic Arch, Atrioventricular Septal Defect, Double Outlet Right Ventricle, Coarctation of the Aorta, Absent Pulmonary Valve Syndrome per Year

eFigure 3. Total Prenatal Detection Rates of Major Congenital Heart Defects

eFigure 4. Prenatal Detection Rates of Congenitally Corrected Transposition of the Great Arteries, Interrupted Aortic Arch, Atrioventricular Septal Defect, Double Outlet Right Ventricle, Ebstein Anomaly, and Pulmonary Atresia by Year

eFigure 5. Prenatal Detection Rates of Persistent Truncus Arteriosus, Absent Pulmonary Valve Syndrome, Tetralogy of Fallot, and Total Anomalous Pulmonary Venous Connection

eFigure 6. First Trimester Screening Period

This supplemental material has been provided by the authors to give readers additional information about their work.

© 2023 Tomek V et al. *JAMA Network Open.*

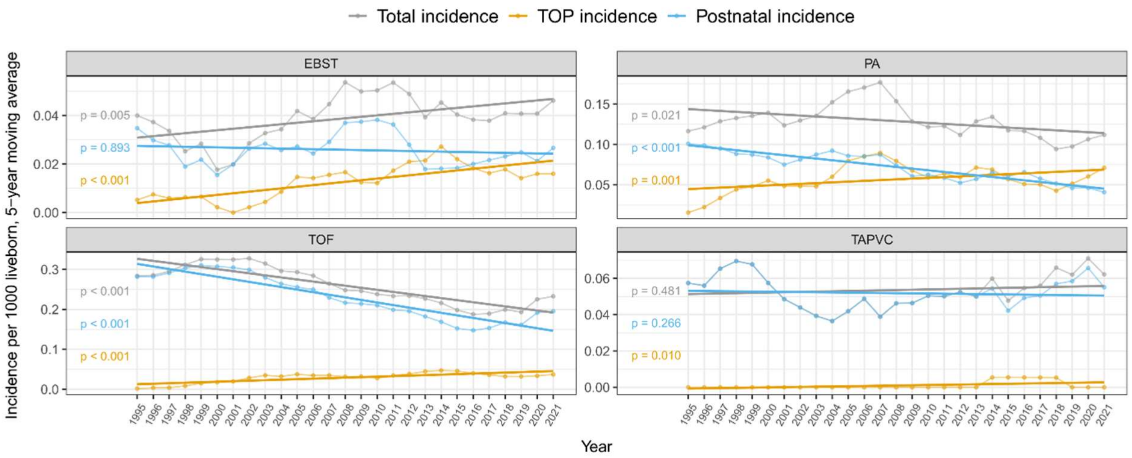

eFigure 1. Total Incidence, Termination Rate, and Postnatal Incidence of Ebstein Anomaly, Pulmonary Atresia, Tetralogy of Fallot, and Total Anomalous Pulmonary Venous Connection per Year

There was a decrease in total and postnatal incidence of and increase TOP incidence TOF (p<0.001). Incidence of all other specific heart lesions remained fairly constant. Five-year moving rates are displayed. Results are plotted at the upper endpoint of the five-year interval. Overall trends estimated by linear regression models.

© 2023 Tomek V et al. *JAMA Network Open.*

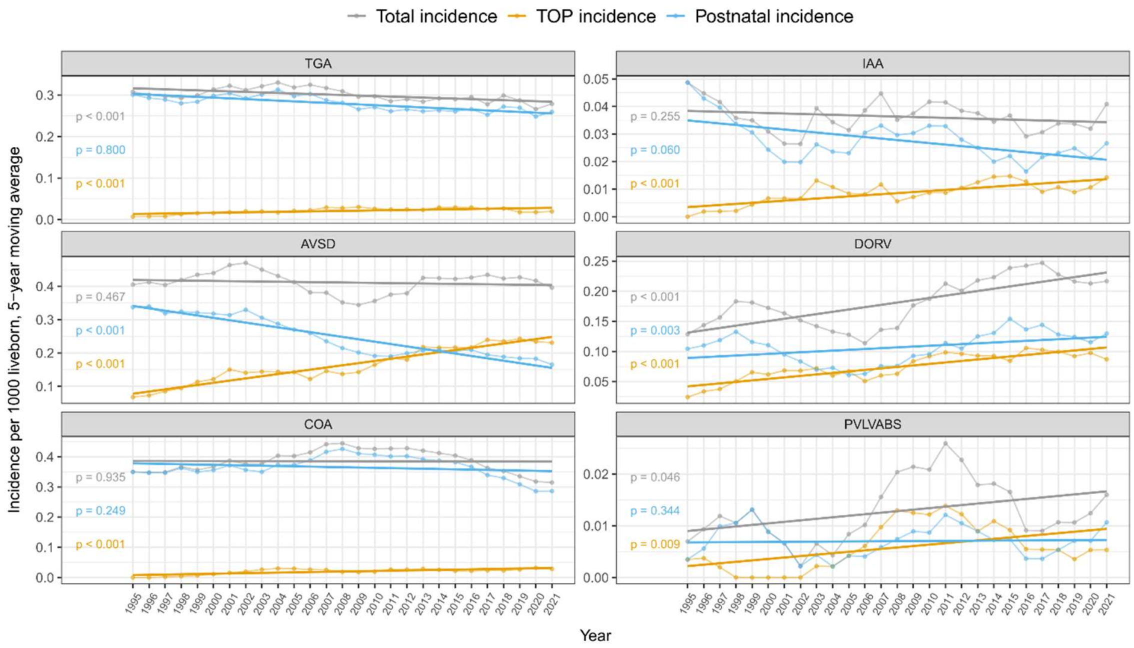

eFigure 2. Total Incidence, Termination Rate, and Postnatal Incidence of Transposition of the Great Arteries, Interrupted Aortic Arch, Atrioventricular Septal Defect, Double Outlet Right Ventricle, Coarctation of the Aorta, Absent Pulmonary Valve Syndrome per Year

There was an increase in total, postnatal and TOP incidence of DORV (p<0.001) and decrease in total incidence and increase in TOP incidence of TGA (p<0.001)CCTGA (p=0.009) and an increase in DORV (0.0002), HLH (p=0.0124), AVSD (p=0.0269) and COA (p=0.0406). Five-year moving rates are displayed. Results are plotted at the upper endpoint of the five-year interval. Overall trends estimated by linear regression models.

© 2023 Tomek V et al. *JAMA Network Open.*

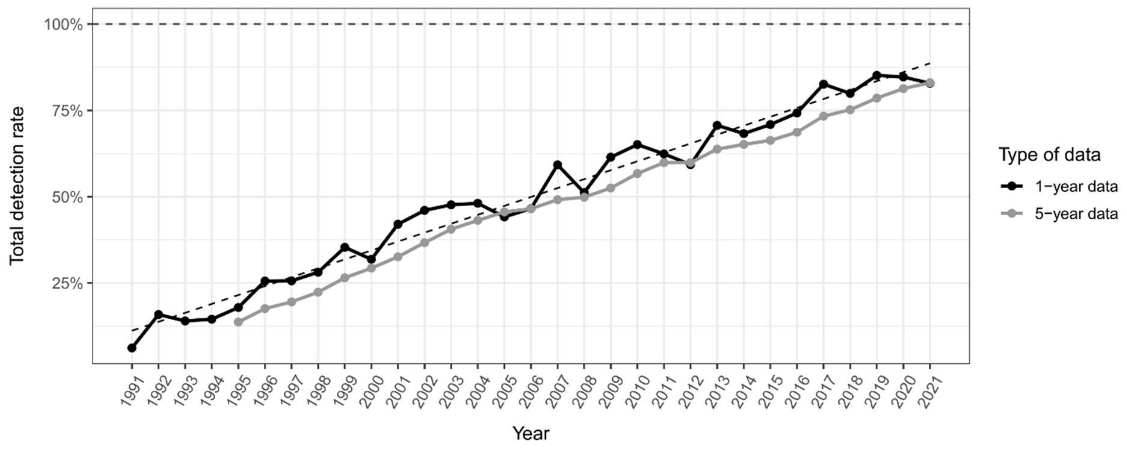

eFigure 3. Total Prenatal Detection Rates of Major Congenital Heart Defects

Black points are based on one year data, gray points are five-year moving rates plotted at the upper endpoint.

© 2023 Tomek V et al. *JAMA Network Open.*

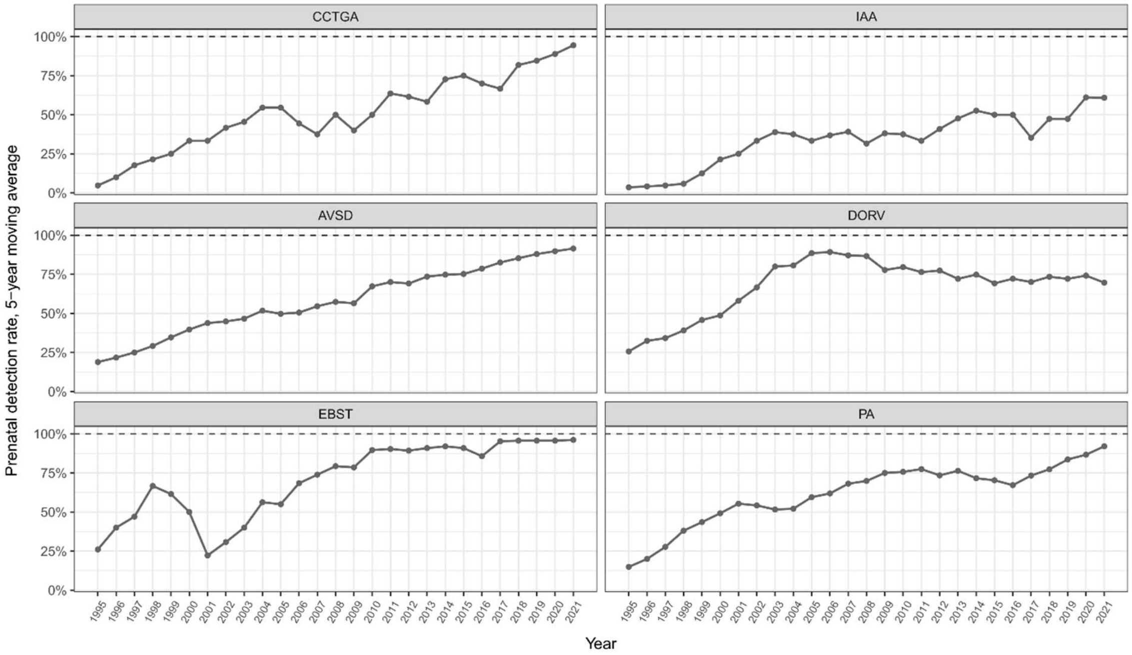

eFigure 4. Prenatal Detection Rates of Congenitally Corrected Transposition of the Great Arteries, Interrupted Aortic Arch, Atrioventricular Septal Defect, Double Outlet Right Ventricle, Ebstein Anomaly, and Pulmonary Atresia by Year

Rates are based on a total number of prenatal (the numerator) and prenatal + postnatal (the denominator) detections of the individual CHD. A detection rate of all individual heart defects was, except DORV, increasing. Five-year moving rates are displayed. Results are plotted at the upper endpoint of the five-year interval to level individual variations in small numbers.

© 2023 Tomek V et al. *JAMA Network Open.*

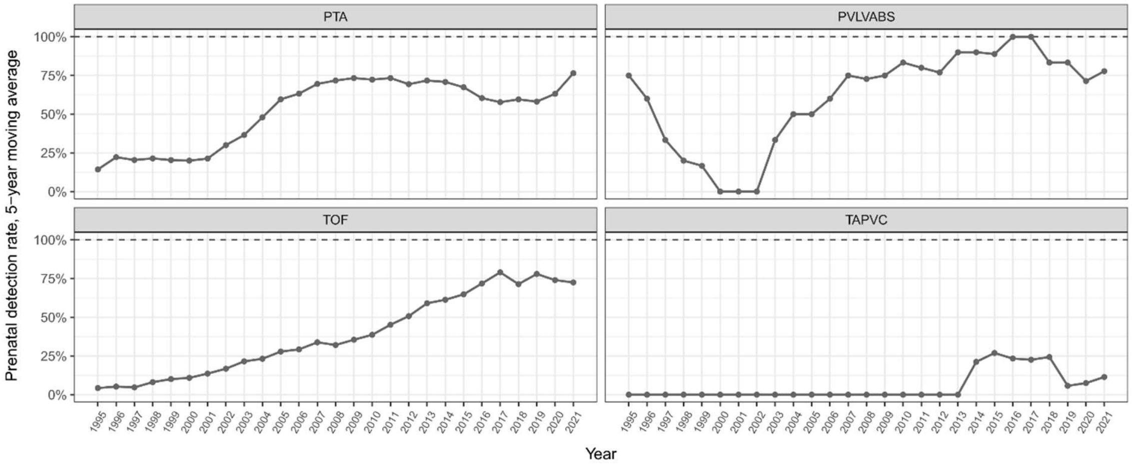

eFigure 5. Prenatal Detection Rates of Persistent Truncus Arteriosus, Absent Pulmonary Valve Syndrome, Tetralogy of Fallot, and Total Anomalous Pulmonary Venous Connection

Rates are based on a total number of prenatal (the numerator) and prenatal + postnatal (the denominator) detections of the individual CHD. A detection rate of TAPVC remained poor. Five-year moving rates are displayed. Results are plotted at the upper endpoint of the five-year interval to level individual variations in small numbers.

© 2023 Tomek V et al. *JAMA Network Open.*

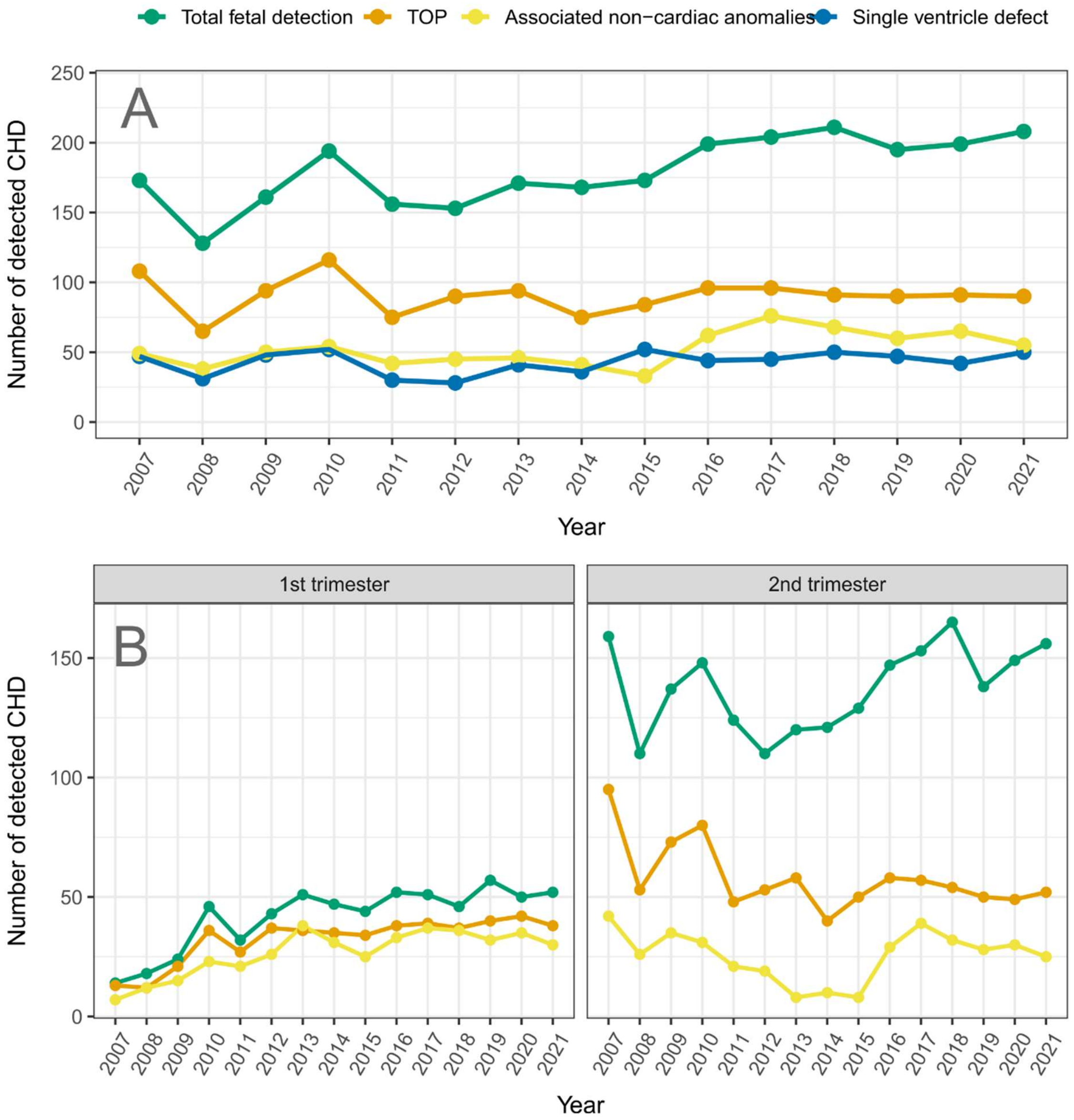

eFigure 6. First Trimester Screening Period

A. Total number of prenatally detected cases shows the overall decreasing trend in TOP rate of all major CHD, pregnancy termination cases (TOP), cases of single ventricle defects and of associated non-cardiac anomalies irrespective of trimester. B. Comparison of total number of prenatally detected cases, pregnancy termination cases (TOP), number of associated non-cardiac anomalies detected in the first and the second trimester, respectively.

© 2023 Tomek V et al. *JAMA Network Open.*
