# Supplementary material for: Pregnancy Termination and Postnatal Major Congenital Heart Defect Prevalence After Introduction of Prenatal Cardiac Screening
Source: JAMA Netw Open. 2023 Sep 15;6(9):e2334069. doi: 10.1001/jamanetworkopen.2023.34069 (PMC10504618; doi:10.1001/jamanetworkopen.2023.34069)
Supplement: Supplement 2. — Data Sharing Statement [file jamanetwopen-e2334069-s002.pdf]

Data Sharing Statement

Tomek. Pregnancy Termination and Postnatal Major Congenital Heart Defect Prevalence. *JAMA Netw Open*. Published September 15, 2023. doi:10.1001/jamanetworkopen.2023.34069

Data
Data available: No

Additional Information
Explanation for why data not available: on request only
